# Clinical effects of wasabi extract containing 6-MSITC on myalgic encephalomyelitis/chronic fatigue syndrome: an open-label trial

**DOI:** 10.1186/s13030-022-00255-0

**Published:** 2022-12-12

**Authors:** Takakazu Oka, Yu Yamada, Battuvshin Lkhagvasuren, Mutsuhiro Nakao, Ryota Nakajima, Masanobu Kanou, Ryuji Hiramatsu, Yo-ichi Nabeshima

**Affiliations:** 1 Department of Psychosomatic Medicine, International University of Health and Welfare Hospital, Iguchi 537-3, Nasushiobara-Shi, Tochigi-Ken 329-2763 Japan; 2 Department of Psychosomatic Medicine, International University of Health and Welfare Narita Hospital, 852 Hatakeda, Narita, Chiba 286-8520 Japan; 3 TEIJIN LIMITED, 2-1, Kasumigaseki 3-Chome, Chiyoda-Ku, Tokyo, 100-8585 Japan; 4 PAL LIMITED, 10-31-105, Furuedai 3-Chome, Suita-Shi, Osaka, 565-0874 Japan

**Keywords:** Myalgic encephalomyelitis/chronic fatigue syndrome, 6-MSITC, Brain fog, Cognitive impairment, Fatigue, Performance status, Clinical effect

## Abstract

**Background:**

Wasabi (*Eutrema japonicum*) is a common pungent spice used in Japan. 6-Methylsulfinylhexyl isothiocyanate (6-MSITC) found in the rhizome of wasabi has been shown to have anti-inflammatory and antioxidant effects, as well as improve neuroinflammation and memory. Therefore, we hypothesized that these effects would be beneficial for treating myalgic encephalomyelitis/chronic fatigue syndrome (ME/CFS). The present study was conducted to investigate the effectiveness of wasabi extract containing 6-MSITC on ME/CFS in an open-label trial.

**Methods:**

Fifteen patients (3 males, 12 females, 20–58 years old) were orally administered wasabi extract (9.6 mg of 6-MSITC/day) for 12 weeks. The following parameters and test results were compared pre- and post-treatment: performance status (PS), self-rating questionnaires, pressure pain threshold (PPT) on the occiput, Trail Making test-A (TMT-A), and hemodynamic patterns determined by an active standing test.

**Results:**

After treatment with 6-MSITC, PS improved significantly (*p* = 0.001). Although the scores on the 11-item Chalder Fatigue scale (CFS-11) and numerical rating scale (NRS) of fatigue did not show significant changes, subjective symptoms improved significantly, including headache frequency (4.1 to 3.0 times/week, *p* = 0.001) and myalgia (4.1 to 2.4 times/week, *p* = 0.019), NRS brain fog scores (5.7 to 4.5, *p* = 0.011), difficulty finding appropriate words (4.8 to 3.7, *p* = 0.015), photophobia (4.8 to 3.5, *p* = 0.008), and the Profile of Mood Status vigor score (46.9 to 50.0, *p* = 0.045). The PPT of the right occiput (17.3 to 21.3 kPa, *p* = 0.01) and TMT-A scores (53.0 to 38.1 s, *p* = 0.007) also changed, suggesting reduced pain sensitivity, and improved cognitive function, respectively. Orthostatic patterns determined by a standing test did not show remarkable changes. There were no serious adverse reactions.

**Conclusion:**

This study suggests that 6-MSITC improves PS as well as subjective symptoms such as pain and cognitive dysfunction, and psychological vitality of patients with ME/CFS. It also improved cognitive performance and increased pain thresholds in these patients. 6-MSITC may be a promising therapeutic option especially for improving cognitive dysfunction associated with ME/CFS.

## Introduction

Myalgic encephalomyelitis/chronic fatigue syndrome (ME/CFS) is a debilitating disease characterized by persistent or relapsing unexplained fatigue that is not relieved by rest, and causes a substantial reduction in daily activities [1]. It is associated with a variety of symptoms such as myalgia, arthralgia, pharyngeal pain, unrefreshing sleep, cognitive impairment (brain fog), and orthostatic intolerance. It often develops suddenly after viral infection and has comorbid diseases such as fibromyalgia and depression. Because of its complexity, effective treatments have yet to be established [2, 3]. Furthermore, while the pathophysiological mechanisms of ME/CFS have not been fully described, the involvement of oxidative stress [4], neuroinflammation [5], and gut microbiota [6] has been postulated.

Wasabi (*Eutrema japonicum*) is a pungent spice commonly used in Japan and is frequently eaten with sushi. In the rhizome of the wasabi plant there is a sulfinyl compound, called 6-methylsulfinylhexyl isothiocyanate (6-MSITC). In laboratory animals, 6-MSITC has been demonstrated to have anti-inflammatory [7, 8], anti-allergenic [9], and antioxidant [10–12] effects, as well as to inhibit neuroinflammation [13]. It has also been shown to improve cognitive function [12, 14]. Given these benefits, 6-MSITC is already sold on the market in Japan as a supplement for improving cognitive function and, to date, no serious side effects have been reported. Based on these findings, we hypothesized that 6-MSITC would be a safe and effective treatment option for patients with ME/CFS. Nakatomi has already conducted a pilot study in which 18 patients with ME ME/CFS took 1 to 3 capsules containing 6-MSITC (1.6 mg per capsule) daily for 1–3 months. Improvements in general conditions were observed in 6 patients and remarkable improvements were observed in 2 patients [15]. This suggests the potential to use 6-MSITC as an effective treatment option for ME/CFS. Unfortunately, however, the dose of 6-MSITC and the duration of administration varied by subject, and the specific effects on each symptom were not examined in that study.

Therefore, to further understand the potential therapeutic benefits, we sought to investigate the effectiveness of 6-MSITC on ME/CFS using a fixed dose (9.6 mg/day) and time period of administration (12 weeks). We also investigated the effects of 6-MSITC on common symptoms of ME/CFS, i.e., fatigue, sleep disturbance, pain, neurocognitive dysfunction, and orthostatic intolerance, in an open-label trial.

## Methods

### Subjects

This open-label trial enrolled adult outpatients with ME/CFS who visited the Department of Psychosomatic Medicine, International University of Health and Welfare (IUHW) hospital and the IUHW Narita hospital and who satisfied the following criteria: (1) insufficient improvement of general conditions three months after conventional treatment (provided at our hospitals to minimize bias due to the natural course and the effect of preceding treatments), (2) between 20 and 70 years of age, and (3) the subject’s performance state (PS) was between 3 and 8 [16, 17]; patients were asked to fill out the questionnaire by themselves and visit the hospital at least once a month. Patients were excluded if their fatigue was due to physical disease such as liver, kidney, heart, respiratory, endocrine, autoimmune, or malignant disease, severe anemia, electrolyte abnormalities, obesity, or pregnancy. Patients were also excluded if they had taken or were currently taking 6-MSITC-containing supplements.

The combination of treatments provided at our hospitals was described previously [18, 19]. In brief, they included (a) instructions on self-pacing and life guidance, e.g. tips to minimize crash and post-exertional malaise (PEM), reduce maladaptive coping behavior as well as improve adaptive and fatigue-relieving behaviors; (b) psychotherapy to reduce guilt, self-blame, worthlessness or helplessness; (c) pharmacotherapy for comorbid physical diseases such as functional gastrointestinal disorders, fibromyalgia, orthostatic intolerance, and psychiatric diseases such as mood and anxiety disorders as well as insomnia; (d) Kampo, traditional Japanese herbal medicines, to improve general condition and energy; (e) treatments to reduce stress and elicit relaxation; and (f) environmental rearrangement at home, the workplace, or school. The combination of interventions varied from person to person, taking into account background factors that may be involved, motivation, energy and cognitive limits.

### Diagnosis of ME/CFS

The diagnosis of ME/CFS was made when patients met the following case definitions and diagnostic criteria: the 1994 Fukuda case definition of CFS [20], the 2005 Canadian clinical case definition of ME/CFS [1], the 2011 International Consensus Criteria for ME [2], the 2015 diagnostic criteria for ME/CFS or systemic exertion intolerance disease [3], and the 2017 Japanese clinical diagnostic criteria for ME/CFS [17]. All patients met these 5 case definitions or diagnostic criteria, with the exception of one subject who was later confirmed to have bipolar disorder after initiation of the study and was subsequently excluded from analysis.

### Procedures

Assessments were made pre- and post-treatment, i.e., on the first and last day of the 12-week 6-MSITC treatment. During this period, patients were asked to take 6 capsules that contained 6-MSITC (1.6 mg per capsule), i.e., two capsules after each meal, for 12 weeks. On the day of assessment, patients underwent medical interview, physical examinations, measurement of mechanical pain thresholds, active standing tests, and blood draws. Thereafter, they filled out questionnaires (described below) and provided narrative impressions of their condition. During the treatment period, we did not change their prescriptions except to prescribe non-steroidal anti-inflammatory drugs as needed. 6-MSITC was provided by Teijin Co. Ltd.

### Measurements

#### Performance status (PS)

Severity of the disease was assessed by the ME/CFS PS, which was determined based on the Japanese case definition of ME/CFS [17]. Their PS was classified from 0 (best performance status) to 9 (worst performance status), and the PS of patients with ME/CFS must be 3 or greater (Table 1).

**Table 1:** The PS score for evaluating severity of fatigue in ME/CFS patients

0: The subject is able to have a normal social life without fatigue and to function without restrictions
1: The subject is able to have a normal social life and work, but often feels fatigue
2: The subject is able to have a normal social life and work, but often needs rest due to general malaise
3: The subject is unable to engage in social life or work for several days a month because of general malaise and needs to rest at home
4: The subject is unable to engage in social life or work for several days a week because of general malaise and needs to rest at home
5: The subject is unable to engage in normal social life or work. The subject is able to do light work, but needs to rest at home several days a week
6: Light work is possible on good days, but the subject rests at home more than 50% of the week
7: The subject is able to take care of him/herself and does not need assistance, but normal social activities and light work are not possible
8: The subject is able to take care of him/herself to a certain extent, but often needs assistance and stays in bed more than 50% of the time during the day
9: The subject is unable to take care of him/herself at all, requires constant assistance, and stays in bed all day

The PS is the degree of fatigue and malaise determined by the Myalgic Encephalomyelitis/Chronic Fatigue Syndrome Clinical Diagnostic Criteria (2017) [17]

*ME/CFS* Myalgic encephalomyelitis/chronic fatigue syndrome, *PS* Performance of status

In a patient with ME/CFS, the condition must be PS 3 or higher

#### Fatigue

Severity of fatigue was assessed by two means, the Japanese version of the 11-item Chalder fatigue scale (CFS-11) score [21, 22] and the numerical rating scale (NRS). The CFS-11 is a well-validated, self-reported scale that measures the physical (questions 1 – 7) and mental (questions 8 – 11) symptoms of fatigue. The maximum score is 33, with higher scores indicating more severe fatigue. The CFS-11 score of the community sample was reported to be 14.2 ± 4.6 [23]. Just prior to initiating 6-MSITC treatment (pre) and during the last seven days of the study (post), subjective fatigue levels (fatigue severity) were also scored by the NRS at 8:00 a.m., 12:00 a.m., 4 p.m., and 8 p.m. for seven consecutive days, because fatigue levels vary over time, with the most severe fatigue a patient could imagine being 10, and the absence of fatigue 0. Severity of fatigue (NRS) was calculated by averaging the 28 NRS scores (4 times/day × 7 days) and these values were compared between pre- and post-intervention.

#### Sleep dysfunction

Sleep dysfunction was assessed using the Pittsburgh Sleep Quality Index (PSQI) [24]. The PSQI is a 19-item self-report questionnaire and the total score ranges from 0 to 21, with a score above 5 indicating poor sleep quality.

#### Pain

Pain-related indices were assessed by subjective reports and by measuring the pressure pain threshold (PPT). Subjective reports on pain included presence (yes or no), frequency (times per week), and severity (NRS) of headache, myalgia, arthralgia, and pharyngeal pain in a week.

The PPT was measured by pressing a digital algometer (Digital Force Gauge, model RZ-20; Aikoh Engineering, Osaka, Japan) perpendicular to acupuncture points GB 20 on both sides of the patient in a relaxed sitting position. The GB20 is located at the junction between the base of the skull and the top of the neck, just lateral to the tendons of the trapezius muscle. The PPT was defined as the minimum level of pressure at which pressure sensation begins to become painful when pressed perpendicularly on the surface of the skin with a digital algometer.

#### Neurocognitive symptoms and function

Neurocognitive indices were assessed by subjective reports on neurocognitive symptoms and the Japanese version of the Trail Making test-A (TMT-A). Subjective reports on neurocognitive symptoms included severity (NRS) of brain fog, difficulty finding words, memory loss, and presence (yes or no) and severity (NRS) of photophobia spanning a week. In the TMT-A, participants were asked to trace the numbers from 1 to 25 as quickly as possible. The time to complete the task and the number of mistakes were recorded. TMT-A has two versions, set A and set B. To minimize any practice effects, half of the participants did set A at the pre-intervention time point and set B at post-intervention and the other half of participants did them oppositely.

#### Orthostatic intolerance

Orthostatic intolerance was assessed by a conventional active standing test. After an adequate resting period of at least 5 min, subjects were asked to lie on a bed in the supine position for another 5 min, followed by attempting to maintain a standing position for a further 5 min. Systolic blood pressure (SBP), diastolic blood pressure (DBP), and heart rate (HR) were recorded every minute by a blood pressure monitoring device (Vital Note, TM-62581, A&D Medical, Japan). The last value in the supine position was accepted as the baseline BP and HR. Based on the results of orthostatic testing, patients were diagnosed into 3 orthostatic intolerance subtypes: normal, postural orthostatic tachycardia syndrome (POTS), and orthostatic hypotension (OH). POTS is characterized by an increase in HR of ≥ 30 beats/min (bpm) or a HR of ≥ 120 bpm on standing without OH and is associated with orthostatic symptoms that are relieved by recumbence [25]. OH was defined by a fall in BP of at least 20 mm Hg in SBP or 10 mm Hg in DBP within 3 min in the upright position [26].

#### Psychological conditions

Psychological conditions were assessed by two self-administered questionnaires, the Profile of Mood States 2^nd^ edition (POMS2) and the Hospital Anxiety and Depression Scale (HADS). The POMS2 evaluates mood states and contains seven scales (anger, confusion, depression, fatigue, tension, vigor, and friendship), as well as total mood disturbance [27]. The T score of each scale was assessed. Negative or positive effects are assumed to be low, average, or high if the scores were 30 – 39, 40–58, or high 60–69, respectively. The HADS evaluates the severity of anxiety and depressive symptoms [28, 29]. The ranges of scores for each subscale are 0–7 for normal, 8–10 for mild abnormalities, 11–14 for moderate abnormalities, and 15–21 for severe abnormalities.

#### Health-related quality of life

To assess the health-related quality of life (QOL), the Medical Outcomes Study Short Form 36 (SF-36) was administered [30]. The SF-36 consists of eight components: physical function, physical role, bodily pain, general health perception, vitality, social functioning, emotional role, and mental health; these are then grouped into three component summaries: the physical component summary (PCS), mental component summary (MCS), and role/social component summary (RCS). Norm-based scores were calculated with the average score for the Japanese population set at 50 points for each subscale. A score below 50 indicates that the function assessed on the subscale is below the Japanese average [30].

#### Safety

The presence of subjective adverse events was asked about at each visit to the hospital. Blood tests were conducted before and after the intervention.

#### Impression

At the last visit to the hospital, patients were asked to describe changes after taking 6-MSITC and their impressions of the treatment.

### Statistical analyses

Results are presented as the mean ± standard deviation (SD). Normality in the data distribution was evaluated by the Kolmogorov–Smirnov test. Differences in continuous variables pre- and post-treatment were assessed by a parametric paired samples Student’s *t*-test or a non-parametric paired samples Wilcoxon signed-rank test, as appropriate. Differences in categorical variables were assessed by the Fisher’s exact test or paired samples McNemar’s test, as appropriate. Effect sizes (ES) for significant differences in the values were calculated using *Cramer’s V, Cohen’s d,* or rank-biserial correlation, as appropriate. Multiple linear regression analysis performed using the enter method was conducted to examine whether changes in predictor variables were associated with changes in PS scores. Statistical significance was set at *p* < 0.05, and all tests were two-tailed. Data were analyzed using SPSS v.21.0 and Jamovi v.2.2.5.

## Results

### Subjects

Twenty patients agreed to participate in the study. Among them, five patients were excluded from the final analysis because they withdrew from participation due to fear that they would not be able to change their drug regimen after the study began (*n* = 2), hospitalization due to disease which was not related to ME/CFS (*n* = 1), mood changes that were subsequently diagnosed as bipolar disorder (*n* = 1), and inability to appear at the hospital on the scheduled day due to quarantine for coronavirus disease-2019 (*n* = 1). Thus, we assessed the data from a total of 15 patients (3 males and 12 females). Their ages ranged from 20 to 58 years (37.5 ± 12.1 years). The mean duration of illness after onset was 5.1 ± 4.0 years. Twelve patients developed ME/CFS suddenly after flu-like episodes (post-infectious type). Five patients had comorbid fibromyalgia.

#### PS

After taking 6-MSITC, PS improved significantly (from 6.8 ± 1.7 to 6.3 ± 2.1, *P* = 0.014, parametric paired samples Student's *t*-test; *P* = 0.026, non-parametric paired samples Wilcoxon signed-rank test). Although PS was unchanged in 9 patients, it showed a decrease in 6 patients (from 8 to 7 in two patients, from 7 to 6 in one patient, from 6 to 4 in one patient, and from 3 to 2 in two patients) after treatment (*P* = 0.001, Fisher’s exact test; Table 2).

**Table 2:** Changes in PS after 6-MSITC treatment

	**Pre**	**Post**	**P**	**ES**
**PS**	(n)	(n)		
9	1	1		
8	4	2		
7	7	8		
6	1	1		
5	0	0		
4	0	1		
3	2	0		
2	0	2		
1	0	0		
0	0	0	**0.001**^a^	0.92
average	6.8 ± 1.7	6.3 ± 2.1	**0.014**^b^ **0.026**^c^	0.73 1

*ES*: effect sizes were calculated using the *Cohen’s d,* rank biserial correlation, or *Cramer’s V* as appropriate. *P*: *p* values were analyzed using ^a^a Fisher’s exact test, ^b^a parametric paired Student’s *t*-test, and ^c^a non-parametric paired Wilcoxon signed rank test. *PS* Performance status; (*n* = 15)

#### Fatigue

Before treatment, the CFS-11 total score was 22.0 ± 6.2, which was higher than that of the community sample [23]. The CFS-11 physical fatigue (from 13.9 ± 4.2 to 14.1 ± 3.3, *P* = 0.884), mental fatigue (from 8.1 ± 3.9 to 6.4 ± 1.8, *P* = 0.063), and total scores (from 22.0 ± 6.2 to 20.5 ± 4.5, *P* = 0.339) did not show significant changes after treatment (paired samples Student's *t*-test). The NRS fatigue score also failed to show significant improvement (from 5.9 ± 2.3 to 5.5 ± 2.6, *P* = 0.150, paired samples Student's *t*-test; Table 3).

**Table 3:** Changes in fatigue-related indices after 6-MSITC treatment

	**Descriptive**	**Pre**	**Post**	**Δ**	**P**	**ES**
CFS-11	Physical	13.9 ± 4.2	14.1 ± 3.3	0.2 ± 5.2	0.884	-0.038
	Mental	8.1 ± 3.9	6.4 ± 1.8	-1.7 ± 3.2	0.063	0.521
	Total	22.0 ± 6.2	20.5 ± 4.5	-1.5 ± 5.7	0.339	0.255
Fatigue	Severity	5.9 ± 2.3	5.5 ± 2.6	-0.4 ± 1.1	0.150	0.393

*Δ*: differences between scores before and after treatment (subtraction of Pre from Post), *ES*: effect sizes were calculated using the *Cohen’s d. P*: *p* values were analyzed using a parametric paired Student’s *t*-test. Severity was described using a numerical rating scale, with 10 being the worst and 0 being none. *CFS-11* 11-item Chalder Fatigue Scale; (*n* = 15)

#### Sleep dysfunction

Before the intervention, 14 of 15 subjects (93.3%) had a PSQI score greater than 5, suggesting that most participants had poor sleep quality. The number of patients whose PSQI score was 5 or less changed from one to three after treatment. However, the mean PSQI score did not change significantly (from 10.9 ± 4.0 to 9.3 ± 3.8, *P* = 0.113, paired samples Student's *t*-test, ES = 0.44).

#### Pain

The frequency of headache (from 4.1 ± 2.6 times/week to 3.0 ± 2.3 times/week, *P* = 0.001) and myalgia (from 4.1 ± 2.7 times/week to 2.4 ± 2.5 times/week, *P* = 0.019), improved significantly. In contrast, the frequency and severity of arthralgia and pharyngeal pain did not change significantly (paired samples Student's *t*-test). The PPT of the right GB 20 increased significantly (from 17.3 ± 10.6 kPa to 21.3 ± 10.6 kPa, *P* = 0.01, paired samples Student's *t*-test), suggesting an increased pressure pain threshold (Table 4).

**Table 4:** Changes in pain symptoms and pressure pain thresholds after 6-MSITC treatment

	**Descriptive**	**Pre**	**Post**	**Δ**	**P**	**ES**
**Symptoms**						
Headache	Yes/No (n)	13/2	12/3		0.317^a^	0.26
	**Frequency**	**4.1 ± 2.6**	**3.0 ± 2.3**	**-1.1 ± 1**	**0.001**^b^	**1.03**
	Severity	5.3 ± 2.8	4.5 ± 3.3	-0.8 ± 2	0.145^b^	0.4
Myalgia	Yes/No (n)	12/3	10/5		0.157^a^	0.36
	**Frequency**	**4.1 ± 2.7**	**2.4 ± 2.5**	**-1.7 ± 2.4**	**0.019**^b^	**0.68**
	Severity	4.9 ± 3.7	3.5 ± 3.4	-1.4 ± 2.7	0.061^b^	0.52
Arthralgia	Yes/No (n)	11/4	7/8		0.157^a^	0.36
	Frequency	3.9 ± 4.6	2.3 ± 2.9	-1.6 ± 3.4	0.091^b^	0.47
	Severity	4.0 ± 3.6	2.8 ± 3.4	-1.2 ± 3.2	0.164^b^	0.38
Pharyngeal pain	Yes/No (n)	9/6	9/6		1^a^	0
	Frequency	2.1 ± 2.4	1.9 ± 2.3	-0.1 ± 2.0	0.803^b^	0.07
	Severity	2.5 ± 2.6	2.5 ± 2.5	-0.1 ± 1.9	0.892^b^	0.04
**PPT** (kPa)	**Right occipital**	**17.3 ± 10.6**	**21.3 ± 10.6**	**4.0 ± 5.2**	**0.01**^b^	**0.77**
	Left occipital	18.8 ± 12.3	19.7 ± 11.5	0.9 ± 7.4	0.633^b^	0.13

*Δ*: differences between scores before and after treatment (subtraction of Pre from Post). *ES*: effect sizes were calculated using the *Cramer’s V* or *Cohen’s d* as appropriate. *P*: *p* values were analyzed using ^a^ a paired samples McNemar’s test and ^b^ a parametric paired Student’s *t*-test. Severity was described using a numerical rating scale, with 10 being the worst and 0 being none. Frequency: days per week; *n* Number; *PPT* Pressure pain threshold of the right and left occipital regions; (*n* = 15)

#### Cognitive dysfunction

The NRS scores, indicating the severity of brain fog (from 5.7 ± 1.6 to 4.5 ± 1.9, *P* = 0.011), difficulty finding appropriate words (from 4.8 ± 2.1 to 3.7 ± 1.7, *P* = 0.015), and photophobia (from 4.8 ± 2.9 to 3.5 ± 3.4, *P* = 0.008) improved significantly, whereas the score for memory loss did not change significantly (paired samples Student's *t*-test). On the TMT-A, the time to reach number 25 was significantly reduced (from 53.0 ± 16.3 s to 38.1 ± 13.8 s, *P* = 0.007, paired samples Student's *t*-test), suggesting improvement in frontal lobe function. As the average time for healthy Japanese subjects is reported to be 29 ± 8 s for people in their 20 s and 30 s and 30 ± 8 s for people in their 40 s, the cognitive function of patients in this study was strongly impaired but showed improvement closer to that of healthy subjects after treatment (Table 5).

**Table 5:** Changes in neurocognitive symptoms and TMT-A test scores after 6-MSITC treatment

	**Descriptive**	**Pre**	**Post**	**Δ**	**P**	**ES**
**Subjective**						
Brain fog	**Severity**	**5.7 ± 1.6**	**4.5 ± 1.9**	**-1.1 ± 1.5**	**0.011**^b^	**0.75**
Difficulty finding words	**Severity**	**4.8 ± 2.1**	**3.7 ± 1.7**	**-1.1 ± 1.5**	**0.015**^b^	**0.72**
Memory loss	Severity	4.0 ± 2.3	3.6 ± 1.8	-0.4 ± 2.1	0.479^b^	0.19
Photophobia	Yes/No (n)	13/2	11/4		0.157^a^	0.36
	**Severity**	**4.8 ± 2.9**	**3.5 ± 3.4**	**-1.3 ± 1.6**	**0.008**^b^	**0.8**
**Cognitive test**						
TMT-A	**Time** (sec)	**53.0 ± 16.3**	**38.1 ± 13.8**	**-14.9 ± 18**	**0.007**^b^	**0.82**

*Δ*: differences between scores before and after treatment (subtraction of Pre from Post). *ES*: effect sizes were calculated using the *Cramer’s V* or *Cohen’s d* as appropriate. *P*: *p* values were analyzed using ^a^ a paired samples McNemar’s test and ^b^ a parametric paired Student’s *t*-test. Severity was described using a numerical rating scale, with 10 being the worst and 0 being none. Frequency: days per week; *n* Number; *TMT-A* Trail Making test-A; (*n* = 15)

#### Orthostatic intolerance

Before treatment, the SBP, DBP, and HR of the 15 patients at baseline was 116.5 ± 16.9 mmHg, 69.5 ± 13.9 mmHg, and 79.7 ± 15.9 bpm, respectively. These baseline values did not differ after treatment. Prior to treatment, two patients could not complete the standing test, one patient with POTS quit the test 1 min after standing and another patient with OH quit the test 2 min after standing. After treatment, although one patient with POTS again quit the test 1 min after standing, another patient with OH completed the test normally. The number (%) of patients that showed either a normal pattern, POTS, or OH did not change significantly (from 10 to 10, from 4 to 5, and from 1 to 0, respectively; Table 6). When the changes in hemodynamic appearance were analyzed individually, 8 patients with a normal pattern and three patients with POTS did not show any change after treatment. However, one POTS patient and one OH patient improved to a normal pattern, whereas two normal patients developed a POTS pattern.

**Table 6:** Changes in hemodynamic patterns determined by an active standing test after 6-MSITC treatment

(a)			(b)		
	**Pre** n (%)	**Post** n (%)	**Pre**	**Changes in patterns** Pre → Post	**Number of patients**
**Completion**			Normal		
Completed	13 (86.7)	14 (93.3)		Normal → Normal	8
Inability to complete	2 (13.3)	1 (6.7)		Normal → POTS	2
**Intolerance type**			POTS		
Normal	10 (66.7)	10 (66.7)		POTS → POTS	3
POTS	4 (26.7)	5 (33.3)		POTS → Normal	1
OH	1 (6.7)	0 (0.0)	OH	OH → Normal	1

(a) The number (%) of patients in each category is shown. (b) Comparison of hemodynamic appearance during an active standing test at pre- and post-treatment periods

*POTS* Postural orthostatic tachycardia syndrome, *OH* Orthostatic hypotension; (*n* = 15)

#### Psychological parameters

On the POMS-2, the vigor score (from 46.9 ± 8.0 to 50.0 ± 10.7, *P* = 0.045, paired samples Student's *t*-test) showed significant improvement, whereas the other scores did not change significantly. On the HADS, neither the anxiety nor depression scores showed significant changes (Table 7).

**Table 7:** Changes in psychological indices after 6-MSITC treatment

	**Descriptive**	**Pre**	**Post**	**Δ**	**P**	**ES**
**POMS-2**	Anger	48.8 ± 11.0	48.0 ± 7.8	-0.8 ± 8.6	0.726	0.09
	Confusion	57.7 ± 10.9	54.8 ± 8.3	-2.9 ± 9.6	0.258	0.3
	Depression	52.3 ± 11.6	51.9 ± 11.2	-0.47 ± 5.0	0.726	0.09
	Fatigue	53.0 ± 8.7	54.4 ± 10.5	1.3 ± 6.7	0.451	-0.2
	Tension	52.3 ± 10.2	50.7 ± 10.3	-1.6 ± 6.0	0.318	0.27
	**Vigor**	**46.9 ± 8.0**	**50.0 ± 10.7**	**3.1 ± 5.4**	**0.045**	**-0.57**
	Friendship	48.7 ± 9.3	52.7 ± 12	4 ± 7.3	0.056	-0.54
	TMD	53.7 ± 9.9	52.1 ± 10.8	-1.7 ± 5.5	0.257	0.3
**HADS**	Anxiety	7.1 ± 4.2	6.3 ± 4.1	-0.8 ± 2.4	0.222	0.33
	Depression	9.0 ± 3.9	7.1 ± 4.6	-1.9 ± 4	0.091	0.47

*Δ*: differences between scores before and after treatment (subtraction of Pre from Post), *HADS* Hospital Anxiety and Depression Scale. *ES*: effect sizes were calculated using the *Cohen’s d. P*: *p* values were analyzed using a parametric paired Student’s t-test. *POMS-2* Profile of Mood States-2, *TMD* Total mood disturbance; (*n* = 15)

#### Health-related quality of life

On the SF-36 subscale, general health perception (from 33.4 ± 6.2 to 36.8 ± 8.1, *P* = 0.036) and vitality scores (from 32.7 ± 7.9 to 35.7 ± 9.0, *P* = 0.039, paired samples Student's *t*-test) improved significantly (Table 8).

**Table 8:** Changes in health-related quality of life after 6-MSITC treatment

	**Pre**	**Post**	**Δ**	**P**	**ES**
**SF-36**					
Physical function	30.4 ± 18.2	29.9 ± 16.6	-0.48 ± 8.9	0.838	0.05
Role physical	24.9 ± 13.1	27.8 ± 9.2	2.9 ± 14	0.442	-0.2
Bodily pain	38.2 ± 12.2	41.1 ± 11.8	2.9 ± 10.8	0.319	-0.27
**General health perception**	**33.4 ± 6.2**	**36.8 ± 8.1**	**3.4 ± 5.6**	**0.036**	**-0.6**
**Vitality**	**32.7 ± 7.9**	**35.7 ± 9.0**	**2.98 ± 5**	**0.039**	**-0.59**
Social functioning	33.4 ± 10.5	34.7 ± 13.1	1.27 ± 15.4	0.754	-0.08
Role emotional	41.4 ± 12.5	41.9 ± 14.7	0.56 ± 8.7	0.808	-0.06
Mental health	43.8 ± 9.3	44.1 ± 10.2	0.37 ± 5.2	0.784	-0.07
PCS	30.5 ± 18.4	32.3 ± 13.9	1.87 ± 8.9	0.435	-0.2
MCS	42.9 ± 9.1	45.3 ± 7.8	2.36 ± 4.8	0.08	-0.49
RCS	40.1 ± 12.3	39.9 ± 11.7	-0.2 ± 10.3	0.941	0.02

*Δ*: differences between scores before and after treatment (subtraction of Pre from Post). *ES*: effect sizes were calculated using Cohen’s *d. P*: *p* values were analyzed using a parametric paired Student’s t-test. *SF-36* Short Form Health Survey, *PCS* Physical component summary, *MCS* Mental component summary, *RCS* Role/Social component summary; (*n* = 15)

#### Safety

No serious adverse events were reported.

#### Impressions of patients

Of the 15 patients, 14 patients felt that the treatment was effective especially for cognitive problems and brain fog, whereas 1 patient stated that they did not feel any positive effects. The impressions of all cases are described in Table 9.

**Table 9:** Impressions on the effects of 6-MSITC treatment by patients with ME/CFS (patient number)

(S1) I used to feel heavy when moving my body, e.g., raising my arms. But now I feel lighter. I feel that gravity has become lighter. I used to have a clogged feeling in my head and my headache became worse after exertion, but now I feel fine even after working for the same amount of time (1 h or so). Previously, I couldn’t find appropriate words and put them together well when I thought about something, and the more I thought about it, the hotter my head and upper back became. But now that I can put together words, the feeling of burning has subsided. In the past, my head was always dull and foggy, but now I have more time when my brain fog is clear
(S2) It was effective for inflammation in my mouth. I always had pain in my mouth due to stomatitis. I also used to feel that my tongue was swollen. But these symptoms have subsided. Post-nasal drip became easier to clear and now it is less congested and stuffy in my throat
(S3) I'm still drowsy and unable to concentrate in the morning, as before, but in the afternoon my head is clearer, and my body is easier to move. I used to be sick in bed for 1 to 4 days when there was a sign of a crash, but now I can recover by bed rest for half a day or several hours. I've become stronger. The pain has changed from general to localized
(S4) I can walk longer than before
(S5) I became a little more active in the daytime. I became able to move a little longer
(S6) In the past, there was always a haze in my head, and I felt like the electrical signal from my brain was cut off and my brain didn’t work. But now, I can put my thoughts together. It was difficult to find words but that has become easier. I became able to make notes in a notebook while reading the material. I became able to concentrate longer on reading books and summarizing materials (from about 10 min to about 1 h). My head is starting to work better although I still have a light fog. I feel like I'm sleeping deeply
(S7) Previously, my head was foggy all the time and my thinking ability and memory were zero, but now I feel like they have become clearer. Compared to when I was healthy, it's still a long way off, but it has improved considerably, and it has become easier to live. I became able to understand conversations and come up with more words. My fatigue lessened from around the second month and the number of days at level 7 has increased from what used to be 8–10. It was a great help!!
(S9) The dullness became better although the time of improvement is still short
(S10) I am more motivated. I have more things I want to do than before. I have an appetite
(S11) I can now do multiple things at the same time such as cooking because my brain fog decreased. Previously, I couldn't remember even 3 or 4 digit numbers, but now I can. I used to be very tired when I woke up with a clear dream, but now I can sleep better with less dreaming. My constipation has improved. I couldn't listen to music because it was mixed with words and noise, but now I can. I couldn’t move my fingers with fine control, but now I can. I no longer collide with people or things when I walk
(S13) I didn't feel clear in my head and couldn't react immediately when someone talked to me, but I can think and react faster than before
(S15) I used to feel strong fatigue. But after taking 6-MSITC, I became less tired
(S16) To be honest, I didn't feel much change
(S17) My frequency of low-grade fever has decreased. My fatigue level was also reduced a little
(S18) I always felt hazy in my brain, and it was challenging, but it became clearer and easier to think about things. By becoming clear, I became able to clearly understand where and how strong and painful my symptoms were, instead of feeling somewhat dull, heavy, and in pain

### Factors that are associated with and predict improvement in PS

**Fig. 1:**
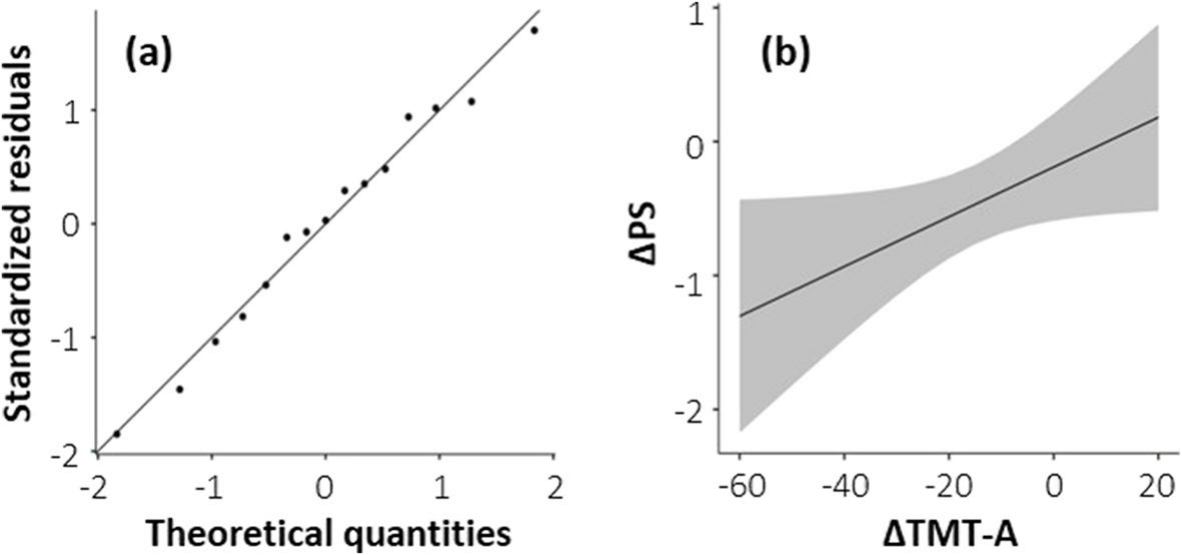
Regression analysis of the ΔPS. **a** Q-Q plot showing the distribution of the residuals. The close alignment between the black dots and the linear trendline demonstrates that the distribution of means across the data is normal. **b** Association between the estimated marginal means of the ΔPS and ΔTMT-A. The trendline and gray areas represent the correlation and 95% confidence intervals, respectively

As shown in Table 10, multiple linear regression analysis indicated that improvements in Trail Making test time (ΔTMT-A) and the POMS-2 tension score were predictive factors for improvement in the PS level (ΔPS) of these patients. This model accounted for 42% of the variance of the ΔPS and was a good fit for the data (F_2_ = 4.4, *p* = 0.038). The distribution of the residuals satisfied the normality assumptions (Fig. 1a). There was no multicollinearity detected between the tested variables (tolerance < 10), no outliers (Cook’s distance < 1; standard residuals <  ± 3.3), and the independence assumption was satisfied (1.5 < Durbin-Watson < 3). The homoscedasticity was not violated by the Breusch-Pagan test (*p* = 0.235). The associations between the estimated marginal means with a 95% confidence interval for the ΔPS and ΔTMT-A are shown in Fig. 1b.

**Table 10:** Multiple linear regression analysis of the changes in ME/CFS performance status (ΔPS) by predictive factors

Predictors	B	Beta	t	*P* value	95% Confidence Interval for Exp (B)		Collinearity Statistics
					**Lower**	**Upper**	**Tolerance**
Model fit: F_2_ = 4.4, p = 0.038; R^2^ = 0.42; Durbin-Watson: 2.44; Heteroscedasticity: 0.235; Cook’s distance: 0.08 ± 0.1							
**Constant**	-0.087		-0.44	0.669	-0.516	0.343	
Δ**TMT-A, time**	0.019	0.523	2.23	0.046	0.001	0.037	1.14
Δ**POMS-2** **Tension**	0.065	0.609	2.59	0.023	0.010	0.120	1.14

*Δ: *differences between scores before and after treatment (subtraction of Pre from Post)*. P: p* values were analyzed using a multiple regression analysis, *TMT-A* Trail Making test-A, *POMS-2* Profile of Mood States-2; (*n* = 15)

## Discussion

The present study demonstrated that 6-MSITC improved PS in ME/CFS patients. Although 6-MSITC did not have significant effects on fatigue and sleep, it did improve subjective symptoms of pain, neurocognitive symptoms, and energy levels in these patients. Along with improvement in these subjective symptoms, 6-MSITC also improved the pressure pain threshold and TMT-A scores.

### PS

It is noteworthy that 6-MSITC improved PS in patients who had not significantly improved after more than 3 months of conventional treatment at our hospitals. The 2017 Japanese clinical diagnostic criteria for ME/CFS [17] requires that the patient have a PS of 3 or higher; therefore, prior to intervention, the PS of all patients was 3 or greater. After 12 weeks of treatment, PS decreased in 6 patients; in 2 patients, PS decreased to less than 3, but the changes in PS of 6 patients were to grades 1 or 2, so not necessarily complete recovery. Further studies are needed to determine if longer-term administration of 6-MSITC can reduce PS even more.

### Fatigue

In contrast to PS, there were no significant changes in CFS-11 scores or NRS scores for fatigue. Prior to conducting this study, we anticipated that 6-MSITC would also improve fatigue. These contradictory results might be attributed, at least in part, to the fact that patients who had been almost bedridden regained some ability to move, perform routine household chores, and begin working. It is reasonable for patients to feel more fatigued, especially after exertion, than when they almost never leave their bed. Such patients recorded higher levels of fatigue three months later, despite being more active. Another explanation could be that fatigue is not the only contributor to PS. In fact, multiple regression analysis demonstrated that the TMT-A and POMS-2 tension scores were predictors of improvement in PS.

### Sleep dysfunction

Unrefreshing sleep is another common symptom of ME/CFS. The present study found that 14 of 15 patients (93.3%) had poor sleep quality, which is comparable to a previous study conducted in Japan that demonstrated that 84 out of 88 (95.5%) patients experienced poor sleep [31]. In the present study, despite one patient (S11) reporting an improvement in sleep quality after 6-MSITC treatment, it did not change PSQI scores significantly. Therefore, the effects of 6-MSITC on sleep seem to be minimal.

### Pain

ME/CFS patients also complain of many pain symptoms, such as headache, myalgia, arthralgia, and pharyngeal pain. Among these, 6-MSITC significantly improved the frequency of headache and myalgia. These effects may be related to an improvement in PPT. The PPT of patients with ME/CFS in this study was lower than that of healthy subjects at the posterior neck region (38.9 – 39.9 kPa) [32], suggesting that patients in this study were more sensitive to pain than healthy subjects. Therefore, the improvement in PPT by 6-MSITC represents a beneficial change for patients with ME/CFS. However, it is unclear why the PPT improved only on the right side. Our previous study also showed a more significant improvement of PPT on the right occiput than left occiput after practicing autogenic training, a relaxation-inducing technique, in healthy subjects [33]. Thus, if 6-MSITC has some relaxing effects, it is reasonable that PPT would improve on the right occiput and reduce the frequency of headaches and myalgia.

### Neurocognitive problems

Neurocognitive symptoms and brain fog are also disabling symptoms for patients with ME/CFS. To our surprise, 6-MSITC treatment greatly improved the cognitive function of patients with ME/CFS in the present study. 6-MSITC improved the subjective severity of brain fog, difficulty finding appropriate words, and photophobia. In accordance with these improvements, many patients (S1, S3, S6, S7, S11, S13, S18) described in detail how their cognitive problems were resolved. The scores on the TMT-A, a frontal lobe function test, were worse in ME/CFS patients than in healthy subjects prior to treatment, but 6-MSITC was found to significantly improve scores. To date, effective treatments for neurocognitive symptoms and brain fog in patients with ME/CFS have been limited. Therefore, 6-MSITC might represent a meaningful treatment option for reliving their symptoms.

### Orthostatic intolerance

Orthostatic intolerance is commonly observed in patients with ME/CFS. On a standing test, some patients may show POTS or OH. The frequency of these orthostatic abnormalities in the present study was almost identical to that in a previous report on Japanese patients [34]. While the present study suggests that 6-MSITC does not have significant effects on general performance in an active standing test, when patients were analyzed individually, one patient who could not complete the standing test prior to treatment gained the ability to remain standing with a normal pattern. Furthermore, one patient with OH and one with POTS also improved to a normal pattern, suggesting improvement in orthostatic intolerance in these patients. In contrast, two patients with a normal pattern prior to treatment developed a pattern of POTS after treatment. Given that the PS of these patients improved, the appearance of POTS must be associated with disease improvement in these subjects. A similar phenomenon had been previously reported [34] in which some patients who showed a normal pattern on a “bad day” exhibited POTS on a “good day”, suggesting that improved condition is associated with the appearance of POTS in some patients. This phenomenon is thought to be due to impaired sympathetic activation on “bad days.” If this is the case, one can hypothesize that impaired sympathetic activation is improved by 6-MSITC treatment, resulting in the appearance of POTS in these patients.

### Psychological conditions and QOL

6-MSITC did not change anxiety or depression significantly. However, this study indicated that 6-MSITC significantly improved energy levels, as evidenced by the vigor score in POMS-2 and vitality score in SF-36. This effect may be related to the patients’ impressions, such as “I became more active (S5)” and “I am more motivated (S10)”, and is thus beneficial for patients with ME/CFS.

### Possible mechanisms

Currently, it is not fully understood how 6-MSITC improves ME/CFS. In vivo and in vitro studies have demonstrated that 6-MSITC has numerous biological effects, such as anti-inflammatory [7, 8], antiplatelet [35], anti-allergenic [9], antioxidant [10–12], and anti-cancer [36, 37]. Moreover, peripheral administration of 6-MSITC also has central effects. For example, it inhibits neuroinflammation caused by intraperitoneal administration of polyinosinic:polycytidylic acid (polyI:C), a viral infection model [13], and has neuroprotective effects on nigral dopaminergic [10] and hippocampal neurons [12]. 6-MSITC also improves cognitive function. For example, in a mouse model of Alzheimer’s disease, systemic administration of 6-MSITC for 10 days ameliorated β-amyloid oligomer (Aβ_1-42_O)-induced memory impairment [12]. Given that it attenuated Aβ_1-42_O-induced hippocampal neuronal cell death [12], hippocampal neuroprotective effects may be involved. In humans, a randomized, double-blind, placebo-controlled trial demonstrated that an eight-week treatment of 6-MSITC improved neurocognitive function, which was assessed by the Stroop Color Word test, in healthy middle-aged and older adults [14].

Oxidative stress [4] and neuroinflammation [5] are thought to be involved in the pathophysiology of ME/CFS. Therefore, one explanation for the beneficial effects of 6-MSITC could be that long-term treatment inhibits oxidative stress and neuroinflammation, thus leading to the improvement of ME/CFS symptoms. Recently, the gut microbiome has been associated with the pathophysiology of ME/CFS, including neuroinflammation and ME/CFS symptoms [6]. Taking this finding into account, another possible mechanism for the effects of 6-MSITC is that long-term oral administration may affect gut microbiota and improve ME/CFS.

### Justification for selection of the 9.6 mg dose of 6-MSITC

We conducted an overdose study in which 10 capsules (16 mg) of 6-MSITC were administered to healthy subjects for 4 weeks to confirm high-dose safety. In a pilot study conducted by Nakatomi et al., they administered 1 (1.6 mg) to 3 (4.8 mg) caps of 6-MSITC daily [15]. Another study conducted by Tanabe et al. administered 9 mg of 6-MSITC to healthy subjects [38]. Based on these studies, we selected a dose of 6 caps (9.6 mg) for administration to our patients. 9.6 mg of 6-MSITC contains approximately 20 g of wasabi (*Wasabia japonica*, Japanese domestic horseradish) [39].

There are several limitations to this study that should be noted. First, this was an open-label trial with a relatively small number of subjects. Therefore, to confirm the effect of 6-MSITC, it will be necessary to compare the changes in these values with a control group in a randomized, controlled trial with a larger sample size. Second, the long-term effects of 6-MSITC are not known yet. However, due to the beneficial effects of 6-MSITC, 10 out of 15 patients continued to take 6-MSITC after this clinical trial ended. Therefore, we will be able to report the long-term effects of 6-MSITC in the near future. Third, the mechanisms of action of 6-MSITC in patients with ME/CFS are not fully understood yet. To address this question, we will be analyzing blood biomarkers from participants in this study. Fourth, we could not assess the effects of 6-MSITC on post-exertional malaise (PEM), one of the hallmarks of this disease. This is partly due to the lack of a simple method to evaluate PEM in a general outpatient setting. The impressions of patient S1 and S3 suggest that 6-MSITC is also effective for PEM, at least in some patients; thus, it will be important to assess this in a future study. In spite of these limitations, this study identified beneficial effects of 6-MSITC on PS, severity of disease, and several symptoms of ME/CFS, including cognitive problems and brain fog. To date, effective treatments for cognitive impairment and brain fog have yet to be established. Therefore, this study suggests that 6-MSITC may represent a promising treatment option for patients with ME/CFS.

## Conclusion

This study demonstrated that 6-MSITC improved PS, frequency of headache and myalgia, neurocognitive symptoms such as brain fog, difficulty finding appropriate words, photophobia, and psychological vitality in patients with ME/CFS. In accordance with the effects on subjective symptoms, it also improved the PPT and scores on the TMT-A. Currently, treatment for neurocognitive dysfunction in ME/CFS patients is lacking. Therefore, 6-MSITC may become a useful treatment option for ME/CFS patients, especially for their neurocognitive symptoms.

## Data Availability

Data sharing is not applicable.

## Abbreviations

Aβ_1-42_O: β-Amyloid oligomers
CFS-11: 11-Item Chalder Fatigue scale
DBP: Diastolic blood pressure
HADS: Hospital Anxiety and Depression Scale
HR: Heart rate
IUHW: International University of Health and Welfare
MCS: Mental component summary
ME/CFS: Myalgic encephalomyelitis/chronic fatigue syndrome
6-MSITC: 6-Methylsulfinylhexyl isothiocyanate
NRS: Numerical rating scale
OH: Orthostatic hypotension
PCS: Physical component summary
PEM: Post-exertional malaise
polyI:C: Polyinosinic:polycytidylic acid
POMS2: Profile of Mood States 2^nd^ edition
POTS: Postural orthostatic tachycardia syndrome
PPT: Pressure pain threshold
PS: Performance status
PSQI: Pittsburgh Sleep Quality Index
QOL: Quality of life
RCS: Role/social component summary
SBP: Systolic blood pressure
SD: Standard deviation
SF-36: Medical Outcomes Study Short Form 36
TMT-A: Trail Making test-A
